# Transcriptome study and identification of potential marker genes related to the stable expression of recombinant proteins in CHO clones

**DOI:** 10.1186/s12896-015-0218-9

**Published:** 2015-10-23

**Authors:** Uros Jamnikar, Petra Nikolic, Ales Belic, Marjanca Blas, Dominik Gaser, Andrej Francky, Holger Laux, Andrej Blejec, Spela Baebler, Kristina Gruden

**Affiliations:** Sandoz Biopharmaceuticals, Kolodvorska 27, SI-1234 Menges, Slovenia; Jozef Stefan Institute, Jamova cesta 39, SI-1000 Ljubljana, Slovenia; Novartis Pharma AG, WKL-681.1.08, 4002 Basel, Switzerland; National Institute of Biology, Vecna pot 111, SI-1000 Ljubljana, Slovenia

**Keywords:** CHO cell line, Stable recombinant protein production, Gene expression, RT-qPCR, DNA microarray, Marker genes

## Abstract

**Background:**

Chinese hamster ovary (CHO) cells have become the host of choice for the production of recombinant proteins, due to their capacity for correct protein folding, assembly, and posttranslational modifications. The most widely used system for recombinant proteins is the gene amplification procedure that uses the CHO-*Dhfr* expression system. However, CHO cells are known to have a very unstable karyotype. This is due to chromosome rearrangements that can arise from translocations and homologous recombination, especially when cells with the CHO-*Dhfr* expression system are treated with methotrexate hydrate. The present method used in the industry for testing clones for their long-term stability of recombinant protein production is empirical, and it involves their cultivation over extended periods of time prior to the selection of the most suitable clone for further bioprocess development. The aim of the present study was the identification of marker genes that can predict stable expression of recombinant genes in particular clones early in the development stage.

**Results:**

The transcriptome profiles of CHO clones with stable and unstable recombinant protein production were investigated over 10-weeks of cultivation, using a DNA microarray. We identified 14 genes that were differentially expressed between the stable and unstable clones already at 2 weeks from the beginning of the cultivation. Their expression was validated by reverse-transcription quantitative real-time PCR (RT-qPCR). Furthermore, the k-nearest neighbour algorithm approach shows that the combination of the gene expression patterns of only five of these 14 genes is sufficient to predict stable recombinant protein production in clones in the early phases of cell-line development.

**Conclusions:**

The exact molecular mechanisms that cause unstable recombinant protein production are not fully understood. However, the expression profiles of some genes in clones with stable and unstable recombinant protein production allow prediction of such instability early in the cell-line development stage. We have thus developed a proof-of-concept for a novel approach to eliminate unstable clones in the CHO-*Dhfr* expression system, which saves time and labour-intensive work in cell-line development.

**Electronic supplementary material:**

The online version of this article (doi:10.1186/s12896-015-0218-9) contains supplementary material, which is available to authorized users.

## Background

The production of recombinant proteins using mammalian cells is a large industry today, which accounts for billions of dollars annually for the production of biotherapeutic products [1]. Chinese hamster ovary (CHO) cells have become the host of choice for recombinant protein production, due to their capacity for correct protein folding, assembly and posttranslational modification. Moreover, CHO cell lines have been well characterised, and they have a very well-known history of regulatory approval for recombinant protein production [2, 3]. The CHO-*Dhfr* expression system is the most widely used for high-level expression of recombinant products in CHO cell lines [1–3].

However, CHO cells are known to have a very unstable karyotype, due to chromosome rearrangements that can arise from translocations and homologous recombination, especially when amplified with methotrexate hydrate (MTX) [4, 5]. In this context, unstable recombinant protein production has been observed in 8 to 63 % of all recombinant CHO (rCHO) cell lines using CHO-*Dhfr*/glutamine synthetase expression systems in the absence and presence of selection pressure (MTX) [6–8]. When the cells are propagated in the absence of a selective agent, the amplified genes can be maintained or lost [9]. Changes in the rCHO cell populations after extended cultivation in the absence and presence of selective pressure have been reported [10, 11]. Fann and colleagues reported unstable recombinant protein production in rCHO cell populations in the presence of MTX, but not to the same extent as when the selective pressure was absent [11]. Previous microarray expression profiling studies have been focused mainly on improved mechanisms underlying high cell productivity [12–16]. Trummer and colleagues identified potential marker genes related to productivity and stress resistance [16]. Some productivity oriented studies have included transcriptomics and/or proteomic studies [17–22].

In the present study, the transcriptome profiles of CHO clones with stable and unstable recombinant protein production were investigated over a 10-week period. The main purpose of the present study was to identify marker genes that can predict stable recombinant protein production in early cell-line development.

## Results and discussion

One of the problems still associated with recombinant protein production using the standard approach of clone screening is, however, the large percentage of clones with unstable productivity of the recombinant protein, which limits rapid and efficient cell-line development. To identify early marker genes for stable recombinant protein production in clones, the transcriptome profiles of the stable and unstable CHO cell clones over a 10-week period were investigated, with two sampling points in the beginning (weeks 1, 2) and two sampling points at the end (weeks 9, 10) of the study period.

### Clone productivity, recombinant gene expression, and copy number in the 10-week study

**Fig. 1:**
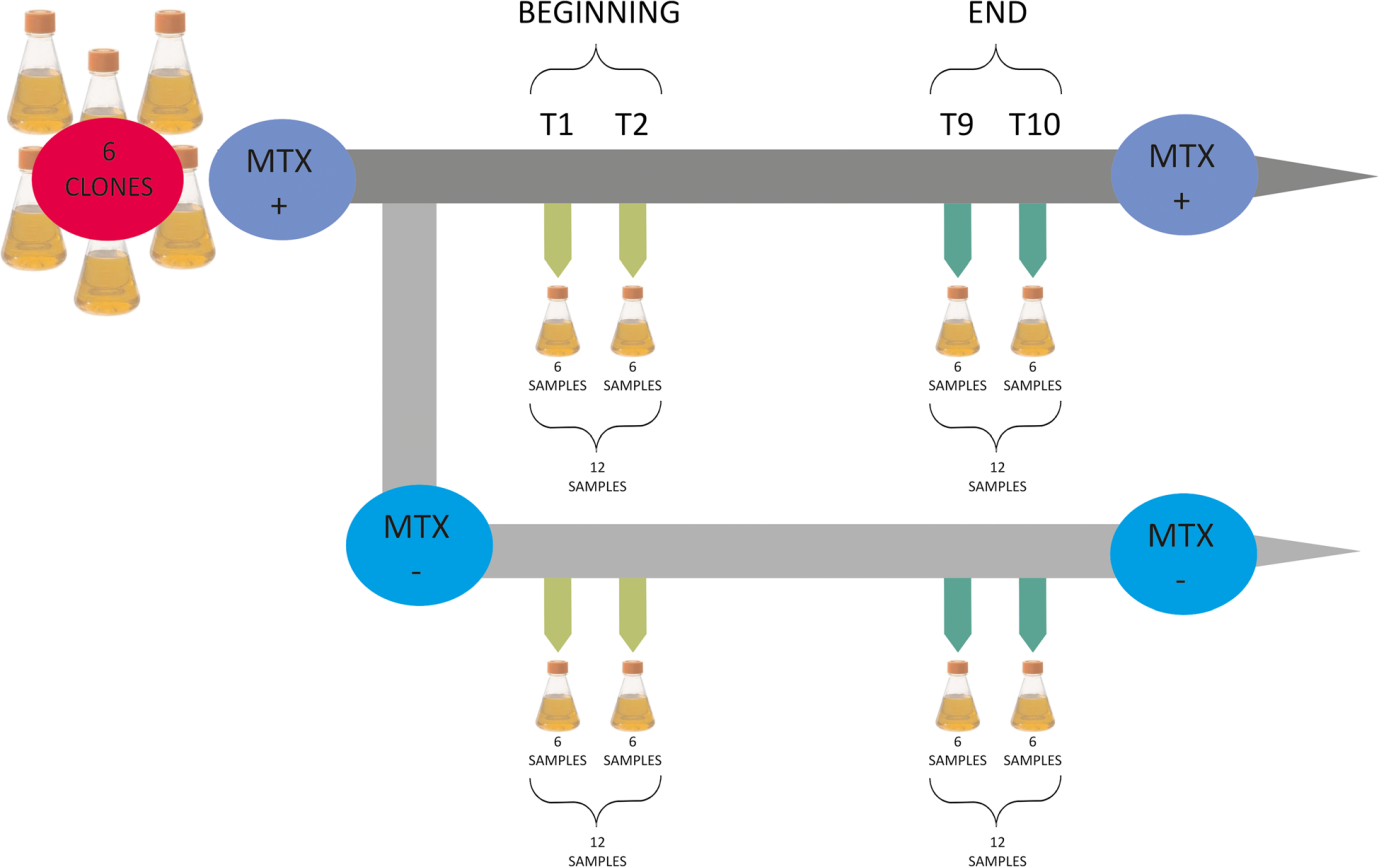
Set-up of the experiment. The study was performed on six transfected clones. Before the stability study began, all six of these clones were grown with MTX in the media to enhance the productivity of the clones. At the beginning of the study, all of the six clones were split and further grown without and with MTX in the media. ‘Beginning’ refers to the beginning of the stability study (sampling at weeks 1, 2) and contains data from the six clones grown without and with MTX in the media, giving 24 samples. Similarly, 24 samples were collected in weeks 9 and 10, and are referred to as the ‘End’ samples

This study was performed on six transfected clones. All six of these clones were grown with MTX in the media before the stability study began, to enhance the productivity of the clones. At the beginning of the stability study, each of these six clones was split and further grown without and with MTX in the media. The ‘Beginning’ refers to the beginning of the stability study. The sampling at weeks 1 and 2 contains data from the six clones (each grown without and with MTX in the media), which resulted in 24 samples. Similarly, 24 samples were collected in weeks 9 and 10, and these are referred to as the ‘End’ samples (Fig. 1).

**Fig. 2:**
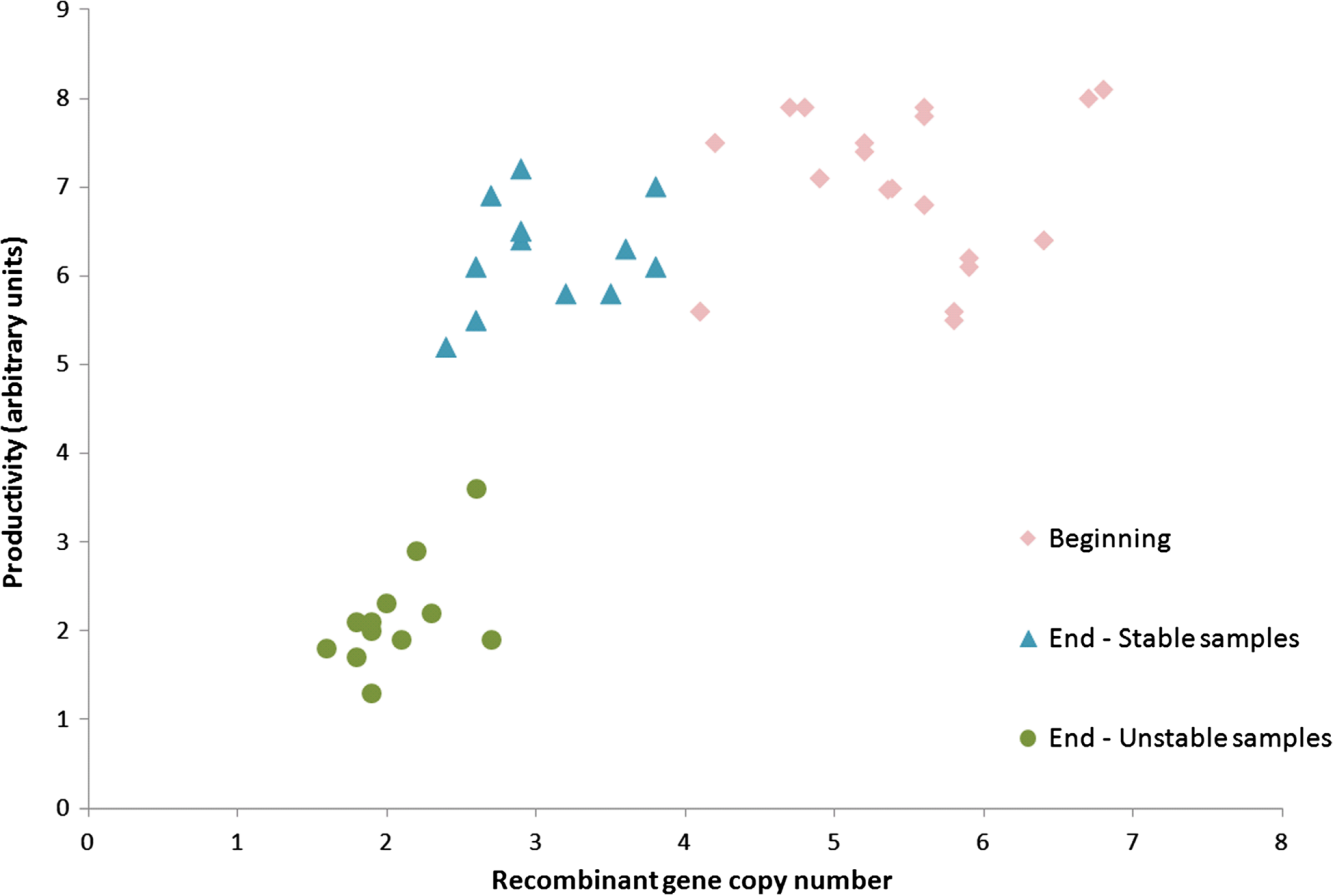
Correlation between the productivity and recombinant gene copy numbers in the clones. The productivity and recombinant gene copy numbers of the clones are represented according to the ‘Beginning’ (weeks 1, 2) and at the ‘End’ (weeks 9, 10) of the 10-week study, as indicated. At the Beginning, when the clones were split for parallel growth without MTX in the media, there were no large differences in the productivity and copy numbers between the clones grown without and with MTX in the media. Due to high biological similarity of several (5) samples at the “Beginning”, some of the data are overlapping, and therefore not all 24 data points are visible

To define the phenotypes of all six of these clones, we measured the productivity and recombinant gene copy numbers for all of the samples at each time point. Thus, the productivity and recombinant gene copy numbers were calculated from the samples grown without MTX (12 samples) and with MTX (12 samples) in the media, from the “Beginning” (2 sampling: at week 1 and week 2), giving 24 samples altogether and similarly at the End (2 sampling: at week 9 and week 10) of the stability study. Two groups of samples were formed at the end of the study, as stable samples (End-stable samples) and unstable samples (End-unstable samples). The relationship between the productivity and the recombinant gene copy number is shown in Fig. 2.

All of the six clones were cultivated in parallel without and with MTX, and therefore we could observe the influence of MTX on the stability of the recombinant protein production. All six of the clones cultivated with MTX in the media were identified as stable producing clones, while the initially high-producing clones cultivated without MTX in the media showed unstable recombinant protein production. The stable producing clones varied on average by 9 % (7.1 to 6.4 arbitrary units) in their initial productivity over the period of 10 weeks. In contrast, the productivity in the unstable clones declined on average by 67 % (7.1 to 2.3 arbitrary units) over the same period. The decline in recombinant gene copy numbers in the stable high-producing clones in the same period was 45 % (5.4 to 3.0 recombinant gene copies per cell), compared to a 61 % decline (5.4 to 2.1 recombinant gene copies per cell) in the unstable clones (Fig. 2).

The unstable recombinant protein production of the CHO-*Dhfr* expression system used in this study over long-term cultivation without selection pressure is attributed to a decrease in the recombinant gene copies [23]. A relation between the decline of recombinant protein productivity and the decline of recombinant gene copy numbers over long-term cultivation was also observed in the present study (Fig. 2). It is common for productivity to drop by up to 71 % when selection pressure is not present in the media, as described by Chusainow [23]. It has been additionally reported that cell lines can lose productivity during cultivation in the absence of selective pressure via transcriptional silencing by methylation, with no loss in recombinant gene copies [24–26]. Promoter methylation is generally known to cause gene silencing, and has recently been shown to additional contribute to unstable recombinant protein production in CHO cell lines expressing an IgG [26]; however, such effects were not prominent in the present study.

### Transcriptional analysis of the stable and unstable clones

Altogether 48 samples (24 stable and 24 unstable samples) were collected and further analysed using a whole-genome DNA microarray and RT-qPCR. The transcript profiles of the stable and unstable clones over their 10-week cultivation were analysed using a whole-genome DNA microarray (Additional file 1). By comparing the transcriptome profile of stable and unstable groups of clones, 295 differently expressed genes were identified (with corrected P value <0.05). As the productivity results for all of the unstable clones were obtained when selection pressure was not present (i.e., without MTX in the media), we have compared these results with the effects of cultivating cells without or with MTX. In all, 199 genes were identified as differentially expressed between the group of clones cultivated without and with MTX in the media (with corrected P value <0.05) and 83 genes were common to both analyses. The remaining 212 genes were specifically differentially expressed when comparing the stable and unstable clones, and were therefore the focus of our further study.

**Fig. 3:**
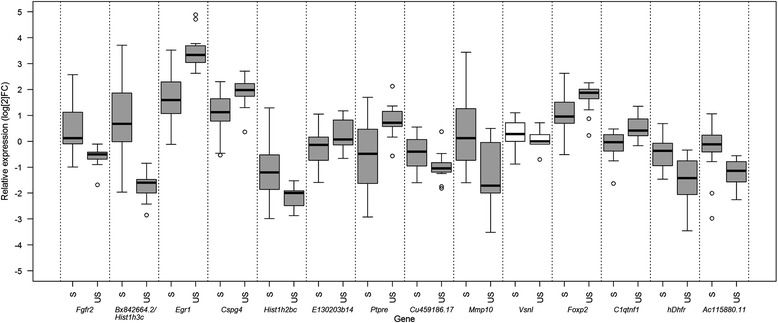
Box plot of the relative gene expression ratios for the stable (S) and unstable (US) clones. The relative gene expression ratios for *Fgfr2*, *BX842664.2/Hist1h3c*, *AC115880.11*, *hDhfr*, *Hist1h2bc*, *Mmp10*, *CU459186.17*, *E130203b14*, *Cspg4*, *C1qtnq*, *Foxp2*, *Egr1* and *Ptpre* (*log*_*2*_FC) for the stable (S) and unstable (UN) clones are shown. The relative expression values were normalised using the geometric mean of the reference genes *Actb/Gapdh.* The threshold for statistical significance between the relative expression ratios of the stable and unstable clones was set to *P* = 0.05 (Welch two tailed t-tests). Box plots for genes where such differences were observed are represented in dark grey, in contrast to the white boxplots for non-significant genes

The expression profiles of the 14 top differentially expressed genes between the stable and unstable clones (where an additional cut-off for a strong fold-change in expression was applied, as: logFC_abs_ >0.8) was verified (Additional file 2) using RT-qPCR. These genes were: *Fgfr2*, *BX842664.2/Hist1h3c*, *AC115880.11*, *E130203b14*, *hDhfr, Hist1h2bc, Cspg4*, *C1qtnq*, *Foxp2*, *Mmp10*, *Vsnl1*, *CU459186.17*, *Egr1* and *Ptpre* (Additional file 3). Among these genes, *E130203B14, BX842664.2/Hist1h3c, Ptpre, Cspg4*, *Fgfr2* and *Vsnl1* were identified as not affected by the presence of MTX in the media. Statistical analysis of the RT-qPCR data comparing the clones that showed stable and unstable recombinant production regardless of the sampling times confirmed differential expression for 13 out of these 14 genes (with corrected P value <0.05) (Fig. 3). The *Vsnl1* gene was the only gene that was not expressed differentially between the stable and unstable clones (with corrected *P* = 0.22) after the RT-qPCR analysis. Seven of the 14 genes tested were up-regulated (*Fgfr2, BX842664.2/Hist1h3c, AC115880.11, hDhfr, Hist1h2bc, Mmp10, CU459186.17*), and six were down-regulated (*E130203b14, Cspg4, C1qtnq, Foxp2, Egr1, Ptpre)* (Fig. 3). The greatest differences in gene expression among these genes specifically for the comparison between stable and unstable clones were observed for the *BX842664.2/Hist1h3c, Ptpre* and *Fgfr2* genes*.*

### Potential marker genes that predict stable recombinant protein production in the early phases of cell-line development

**Fig. 4:**
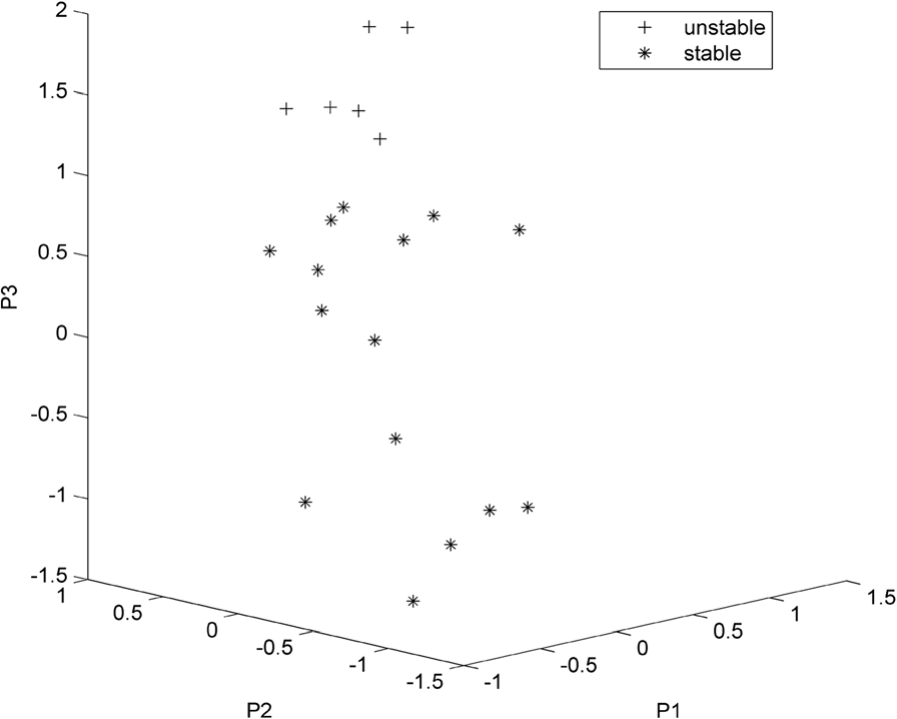
Three-dimensional representation based on principal component analysis. The properties of the clones are described according to the first three principal components (P1, P2, P3) of the expression of the genes *E130203B14, BX842664.2/Hist1h3c, Ptpre, Cspg4* and *Fgfr2*

The main purpose of the present study was to identify marker genes in the early phases of cell-line development that can be used to predict stable recombinant protein production. Thus, we only used the data from the samples collected at the beginning of the study (i.e., ‘Beginning’), without or with MTX in the media, to determine whether their expression profiles could be used to separate them into stable and unstable clones as determined at the end of the study (i.e., ‘End’). Five genes (*E130203B14, BX842664.2/Hist1h3c, Ptpre, Cspg4*, *Fgfr2*) were selected for this analysis from among these 13 differentially expressed genes because the expression of these genes was shown not to be affected by the presence of MTX in the media and all five genes were differently expressed between stable and unstable samples. Three out of the 24 samples used here did not pass our quality control criteria for reliable quantification by RT-qPCR for all of these five genes, and these were thus excluded from analysis. Principal component analysis (PCA) of the expression of these five genes shows that more than 92 % of the total variability of the gene expression data can be explained by the first three principal components. When the first three principal components were presented in three-dimensional form (Fig. 4), there was clear separation of the stable and unstable clones. The classification with the k-nearest neighbour algorithm also confirmed the separation of the two groups. For the Beginning data (i.e., those originating at the beginning of the study), the optimal number of clusters is four where the unstable clones form a distinct cluster. Even for a sub-optimal number of clusters (three and five clusters), the unstable clones form a separate cluster, which suggests that unstable clones form a very compact and distinctive cluster. By observing the sample grouping in Fig. 4, it is evident that the data can be grouped into several clusters with the unstable clones as a separate cluster.

Numerous approaches have been addressed to predict stable recombinant protein production in early cell-line development. For example, Dorai and colleagues [27] identified apoptosis as a possible cause of instability using a flow cytometry method for the identification of apoptotic cells in early cell-line development (i.e., caspase-3 activity). Barnes and colleagues [28] indicated that the levels of recombinant mRNA expression detected by Northern blotting related to stable recombinant protein production at the beginning of long-term cell culture. A whole transcriptome marker identification approach as described in the present study has not been described previously.

### Putative functional correlations of potential marker genes with stable recombinant protein production

Genes *Fgfr2* and *Egr1* of the identified potential markers of instability have functions related to cell growth [29–32], genes *hDhfr*, *Hist1h2bc* and *BX842664.2/Hist1h3c* are involved in DNA replication and chromosomal stability [33–36]. The following two genes *Cspg4* and protease *Mmp10* are important in cell migration and cell regulation [37–42]. Another group of potential marker genes (*Ptpre and Vsnl1*) is involved in intracellular signaling [43–47]. Genes *Foxp2 and Ac115880.11* are regulators of transcription [48–50] and *C1qtnf1* is involved in regulation of gene expression [51–53]. All (but Foxp2 and *Ac115880.11*) were already implied in relation to cancerogenesis showing the potential link of identified genes to instability of cellular processes in mammals. No functions for genes *E130203b14* and Cu459186.17 have been described to date.

## Conclusions

The precise molecular mechanisms behind genetically unstable transgene expression are not fully understood. However, some gene expression properties between clones with stable and unstable recombinant production can be used to predict this instability. By combining the expression patterns of the *E130203B14*, *BX842664.2/Hist1h3c*, *Ptpre*, *Cspg4* and *Fgfr2* genes using principal component analysis, clear separation of the stable and unstable clones can be achieved through observing just the first three most significant principal components. Based on this analysis, the unstable clones in our setting can already be excluded in the early stage of such cell-line development. In this way, a lot of labour-intensive work, and in particular a lot of time, can be saved in the development of such cell lines. However, potential marker genes were identified for a limited number of clones, and thus this approach needs to be tested further to be fully implemented as a replacement for the standard methods of screening stability.

## Methods

### Cell line development

The CHO-der cell line was originally purchased from the European Collection of Cell Cultures (ECACC). The CHO-der1 cell line used in the present study was developed from the CHO-der cell line without any genetic modifications, at Sandoz (Cell and Molecular Biology Department, Slovenia). It was adapted for in-house serum-free media and for better growth performance.

The expression vector was constructed in GeneArt (Life Technologies), and it consisted of the recombinant gene plus the antibiotic resistance and recombinant *Dhfr (rDhfr)* gene. The linearised expression vector was transfected to the host CHO cell line using the nucleofection programme for CHO cells (Nucleofector, Lonza), according to the manufacturer protocol. After transfection, antibiotic selection was performed, followed by amplification of the recombinant gene by MTX. The cloning was performed using ClonePix FL technology (Molecular Devices).

### Experimental set-up

Six high-producing clones were selected for this study. All of these six clones were grown in two parallel settings: without MTX in the media, and with MTX in the media. The in-house serum-free media was supplemented with L-glutamine and was used for all of these cultures. The cultures were propagated in suspension and diluted to achieve initial cell concentrations of 2.0–3.0 × 10^5^ cells/ml. The cells were cultivated for 10 weeks, with passages performed twice per week. The samples for RNA and DNA isolation were taken on day 3 (at mid-log phase) of weeks 1, 2, 9 and 10. In all 48 samples were collected in the study (6 clones grown without and with MTX in the media), each sample originating from 4 data points (weeks 1, 2, 9 and 10). The cells were counted during the passages twice per week using Vicell (Beckman Coulter). To measure the productivity, batches were started from each clone on weeks 1, 2, 9 and 10. The productivity was measured by Octet (Forte Bio), which uses the bio-layer interferometry technology for monitoring the binding of proteins to their partners directly in real time. To confirm that the clones under investigation varied only in terms of the absence and presence of MTX, the cultures were cultivated in parallel under the same conditions (37 °C, 10 % CO_2_, 30 ml working volume, in shaking flasks).

### RNA and DNA isolation

The total RNA was isolated using the automated QiaCube (Qiagen) system with RNeasy mini kits (Qiagen), following the manufacturer protocol. The total RNA was examined using an spectrophotometer (ND-1000; NanoDrop Technologies), with the total RNA integrity monitored using an RNA nano-chip on a Bioanalyser 2100 (Agilent). The same total RNA was used for the DNA microarray and for the RT-qPCR analysis. An additional step of genomic DNA removal using DNase I (Ambion) was performed prior to the cDNA synthesis. After the DNase I treatment, the RNA was transcribed into cDNA using SuperScript VILO kits (Invitrogen), according to the manufacturer protocol. The genomic DNA (gDNA) from the samples was isolated using DNA Blood kits (Qiagen), with an automated system for DNA isolation (QiaCube, Qiagen), according to the manufacturer protocol. The gDNA was quantified using spectrophotometer (ND-1000; NanoDrop Technologies).

### Microarray hybridisation

The proprietary CHO-specific DNA microarray (Affymetrix) used in this study consisted of 61,223 probe sets, which targeted approximately 26,227 Chinese hamster unique gene IDs, and 14,657 unique Ensembl mouse genes. Before being hybridised to the microarray, all of the mRNA samples were diluted to the same concentration (50 ng/μl). Biotinylated cRNA was prepared according to the protocol described in the Affymetrix technical manual. The subsequent hybridisation was performed in a GeneChip Hybridisation oven 640 (Affymetrix), and the processing was carried out using a GeneChip Fluidics station 450 (Affymetrix).

### Microarray data processing and analysis

The studied clones from all four data points (week 1, 2 – ‘Beginning’ and week 9, 10 – ‘End’) were divided into two main groups: clones with stable recombinant protein production (24 stable clones from all data points), and clones with unstable recombinant protein production (24 unstable clones from all data points). The clones were classified here as unstable if there was a decrease in their productivity of >30 % and if the transgene copy number dropped by >50 % over the study period of 10 weeks.

The raw image files were processed using the GeneSpring GX software (Agilent Technologies), and normalised using the robust multichip average algorithm [54–56]. All of the further statistical analysis was performed in the Bioconductor using the *limma* package [57, 58]. To reduce the extent of false positive results, the non-expressed genes were filtered out (i.e., those with expression value below background in at least 80 % of all samples). Empirical Bayes modelling that took into account the stability and the presence of MTX was used to detect differentially expressed genes between the different clones ([59]; for corrected P ≤0.1). Only 524 probes (out of 61,223 probes) were used for the further analysis. The number of transcripts were further reduced to 14 genes (logFC_abs_ >0.8; for corrected *P* <0.05).

### Quantitative real-time qPCR

Based on DNA microarray data, 14 genes that were differentially expressed between the stable and unstable clones were chosen for further verification using RT-qPCR. The primer pairs and probes were designed in the region of the microarray oligo design, to ensure compatibility of the data between both of the platforms. The TaqMan-MGB® probes for all of the genes were designed and manufactured as Custom TaqMan Gene Expression Assays, by Life Technologies. For relative expression calculations, two reference genes were used (*Actb*, *Gapdh*). The primers and probes used for the assays (i.e., gene expression and copy number) are listed in detail in Tables 1 and 2. The QIAgility automated liquid-handling system (Qiagen) was used to prepare two cDNA dilutions (30×, 300×) per sample, and to pipette the cDNA samples and master mix into the 384-well plates. The optimal dilution factors were determined individually for each amplicon on a subset of samples, for the quantification cycle (Cq) values to be in the range of 22–34. All of the RT-qPCR reactions were performed in triplicate on an ABI PRISM® 7900 Sequence Detection system (Life Technologies), in 384-well plate format using universal cycling conditions. Each sample was analysed as two dilutions and three replicates per dilution step. The only samples used for the relative quantification where those where the ΔCq between the two dilutions of the target gene did not deviate by more than 0.5 from ΔCq of the reference gene.

**Table 1:** List of the primers and probes of the reference genes used in the RT-qPCR analysis

Gene Name	Forward Primer (5′ → 3′)	Reverse Primer (5′ → 3′)	Probe (5′ → 3′)
Reference gene for copy number			
*Glucagon (Gluc)*	ATTGCCAAACGCCACGAT	CCAAGCAATGAATTCCTTTGC	CTGAAGGGACCTTTACCA
Reference genes for gene expression			
*Actb*	AGCCACGCTCGGTCAG	CATCCTGCGTCTGGACCT	CCGGGACCTGACAGACT
*Gapdh*	TCAACGGGAAGGCCATCAC	CCATTTGATGTTGGCGGGATC	TCGCTCCTGGAAGATG
*Transgene*			
*rDhfr*	ATATGGGGATTGGCAAGAACG	CATTCTTTGGAAGTACTTGAACTCGTT	AGACCGACCCTGGC

The reference genes were used for the copy number and gene expression calculations. All of the primers and probes were designed as TaqMan Gene Expression Assays labelled with FAM (3′) and MGB (5′). The sequence of the recombinant gene is confidential

**Table 2:** List of the primers and probes of the 14 potential marker genes used in the RT-qPCR analysis

Gene Symbol	Forward Primer (5′ → 3′)	Reverse Primer (5′ → 3′)	Probe (5′ → 3′)
*Fgfr2*	GCCTGAGTTACACATCCATCACA	GATGATGAAGGTCCTGAAGCTGTTA	TTGGCCTCACATCTCC
*BX842664.2/ Hist1h3c*	GGCCCAGACATGGACACT	CCATGAGGCACTGGGACTTT	AAGCGCCCCATCAGC
*AC115880.11*	CGAGCTTTTCACCAGTAGAGATAGTTA	TTGACACATACAGCTCCAATTCCA	ACGGGCTTCAGTCTTC
*E130203B14*	CCAGTGGGTACATCACATGAGAGA	CCCGAGTGGGAGCTGACT	AAACTGTGCCAAACTC
*hDhfr*	ATATGGGGATTGGCAAGAACG	CATTCTTTGGAAGTACTTGAACTCGTT	AGACCTACCCTGGCCT
*Hist1h2bc*	ACGAGGAGTAGACCTGATGATGT	GTATCACCTATTTCCATTGTCTCAATTGC	CAGTGCTGGACGTTGTT
*Cspg4*	GCCATGTGGCCTAGCTTCAT	AAACAGGTGAGAATAGAGGACTTTGG	CAAGCTCTTGAATTCC
*C1qtnf1*	CATTCCACAGACACTGGATGGA	GCCAAAGAAGCCAGGACTGA	CTGACCCCATCATCCC
*Foxp2*	GGGCTTACGGCTTATACTCTATGTG	CCCAGTTAGTGGTAATTCTATCAAGTACTTT	ACGGTGCCATGAATCC
*Mmp10*	CAGGAATCGAGCCACAAATTGATG	TCAAACTGTGATGATCCATGGAAGAA	AATGCCTGCAACACCG
*Vsnl1*	ACCCTTAAGCATATGTCTTTGGAATTTGA	TTCCGAAATGAACAAATCGTCTGTT	TCATCCAGCCCCTCCC
*CU459186.1*	GGGAGGCCGGTTTTGG	TTGTGCAACACCCAGAGACTAC	TTGCTGCCCGGTATCC
*Egr1*	GCTCACCTCTGGCCTTAAAGG	CATTCTGGAGAACCAAAGCT	CAGCTCAGCCCTCTTC
*Ptpre*	CCCTCCAGTCTCTTGGCTAATG	GCAAACTGAGTCTCTGTGTCTTAGG	CCACAACCAAATTCAG

All of the primers and probes were designed as TaqMan Gene Expression Assays labelled with FAM (3′) and MGB (5′)

### RT-qPCR data processing and statistical analysis

The SDS 2.1 software (Life Technologies) was used for fluorescence acquisition and Cq calculation. For this calculation, the baseline was set automatically and the fluorescence threshold was set manually (0.1) to intersect with the linear part of the amplification curves of all of the amplicons in all of the runs. For the statistical analysis, the relative quantification approach was used [60]. The geometric means of the Cq values of all of the reference genes were used as the final reference gene values [61]. Relative expression was calculated separately for each dilution of each sample, and averaged, to yield the final relative expression for the samples. Welch two-tailed t-tests [62] were used to determine the statistically significant differences between the relative expression ratios of each transgene in the stable and unstable samples, with corrected *P* = 0.05 as the limit for statistical significance.

The copy numbers of each recombinant gene were calculated using the absolute quantification method. A standard curve was constructed using the DNA of the same expressed vector as was used for the transfection of the host cell line and gDNA of the parental CHO-der1 derived host cell line. The Cq calculations were performed as described above. The copy numbers of the recombinant gene and endogenous gene (*Gluc*, *Glucagon* gene) in the samples were extrapolated from the standard curves. The ratios between the endogenous gene *Gluc* (single copy gene) and the recombinant gene were calculated, to determine the transgene copy numbers per cell.

### PCA analysis and three-dimensional representation

The properties of the clones described by the expression of the five most specifically expressed genes between the stable and unstable samples were visualised in MATLAB2014 (The Mathworks Inc.). Principal component analysis was performed on the expression data (RT-qPCR data) of the five most differently expressed genes which were not affected by MTX, and the three most significant principal components are presented three dimensions [63]. To systematically evaluate the separation of the stable and unstable clones, the k-nearest neighbour clustering algorithm was used for the three-dimensional representation [64]. This algorithm uses unsupervised learning, where the goal is to separate the data into a predefined number of clusters, while no information on cluster membership of each training sample is provided. The algorithm minimizes the sum of the squared Euclidian distances between the members and the centroids of the clusters. The algorithm clusters the data into a number of predefined clusters, where no information on cluster membership is provided in advance. An optimal number of clusters can be identified by observing the total sum of all of the sample distances to the cluster centres. The optimal number of clusters is found by analysing a curve defined by the total sum of distances versus the number of clusters. A distinctive change in a slope in the curve defines the optimal number of clusters. As a result, the most likely class centroids and its members are estimated. The rate of correctly classified samples was taken as a measure for the separation of the stable and unstable groups on the basis of the gene expression data of five genes. The k-means function of MATLAB2014 (The Mathworks Inc.) was used for this task.

### Availability of supporting data

The data sets supporting the results of this article are included within the article (and its additional files).

## Additional files

Additional file 1:
**MA-plot of the DNA microarray data.** The MA-Plot represents the DNA microarray data that were background corrected and RMA normalised. For differential gene expression, the eBayes linear modelling method was used. Log A (X-axis), logarithm of the average gene expression; Log2 fold-change (y-axis), log2 ratio between gene expressed in the stable versus the unstable clones for all four of the data points (weeks 1, 2, 9, 10). (TIFF 436 kb) Additional file 2:
**Correlation between gene expression for the DNA microarray and the RT-qPCR.** Correlation for the expression of the 14 potential marker genes for stability between the DNA microarray and the RT-qPCR. (TIFF 41 kb) Additional file 3:
**Names and biological functions of the 14 potential marker genes.** Short and full gene names including biological functions of the 14 potential marker genes. (XLSX 13 kb)

## Abbreviations

CHO: Chinese hamster ovary
MTX: Methotrexate hydrate
Dhfr: Dihydrofolate reductase
RT-qPCR: Reverse-transcription quantitative real-time PCR
rCHO: Recombinant CHO
IgG: Immunoglobulin G
*hDhfr*: Hamster (*Cricetulus griseus*) gene encoding dihydrofolate reductase
rDhfr: Recombinant *Dhfr* gene
PCA: Principal component analysis
CHO-der1: Derivate of publicly available CHO cell line
FC: Fold-change
Cq: Quantification cycle
gDNA: Genomic DNA
