# Supplementary figures and images for: Transcriptome study and identification of potential marker genes related to the stable expression of recombinant proteins in CHO clones
Source: BMC Biotechnol. 2015 Oct 23;15:98. doi: 10.1186/s12896-015-0218-9 (PMC4812793; doi:10.1186/s12896-015-0218-9)

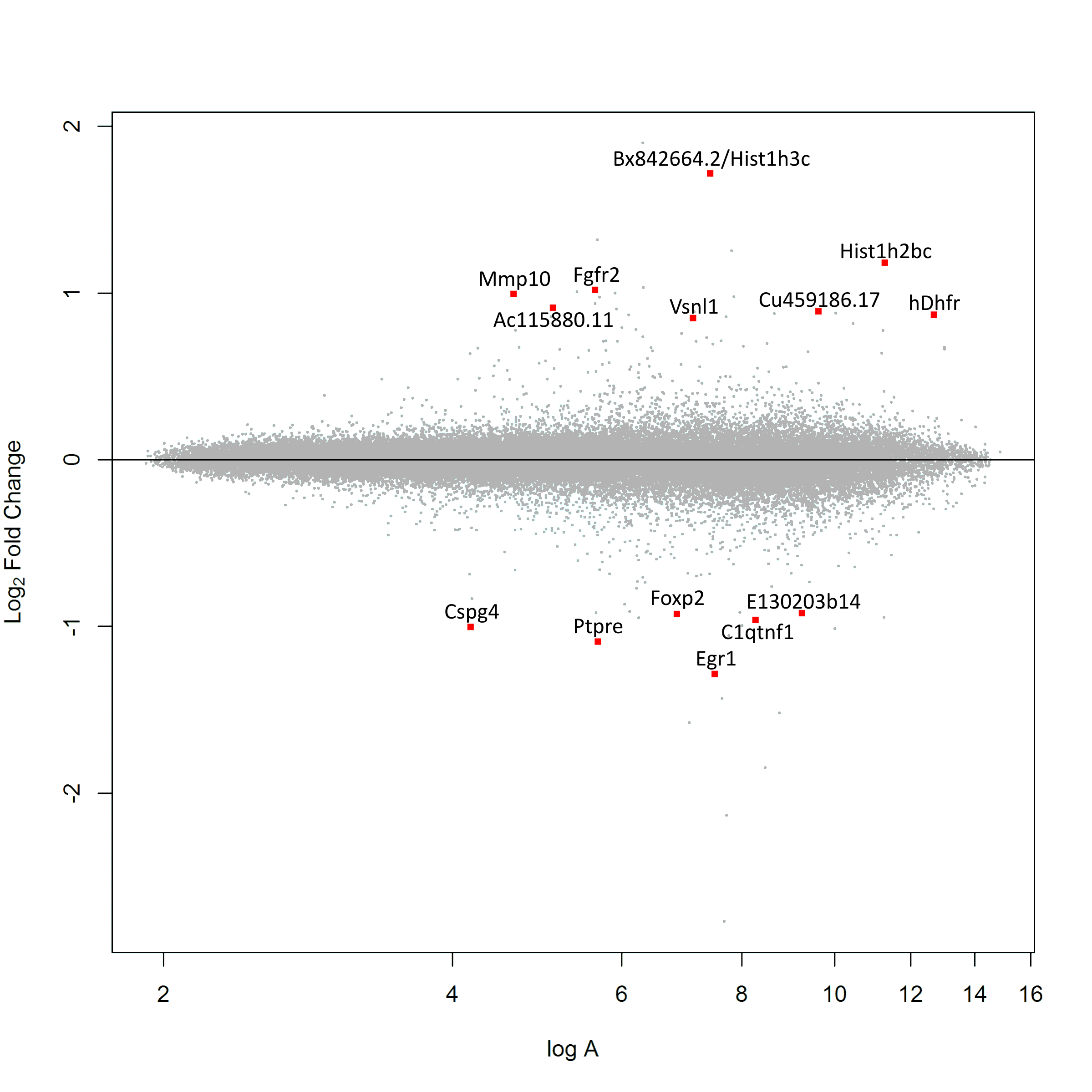

Supplement: Additional file 1: — MA-plot of the DNA microarray data. The MA-Plot represents the DNA microarray data that were background corrected and RMA normalised. For differential gene expression, the eBayes linear modelling method was used. Log A (X-axis), logarithm of the average gene expression; Log2 fold-change (y-axis), log2 ratio between gene expressed in the stable versus the unstable clones for all four of the data points (weeks 1, 2, 9, 10). (TIFF 436 kb) [file 12896_2015_218_MOESM1_ESM.tiff]

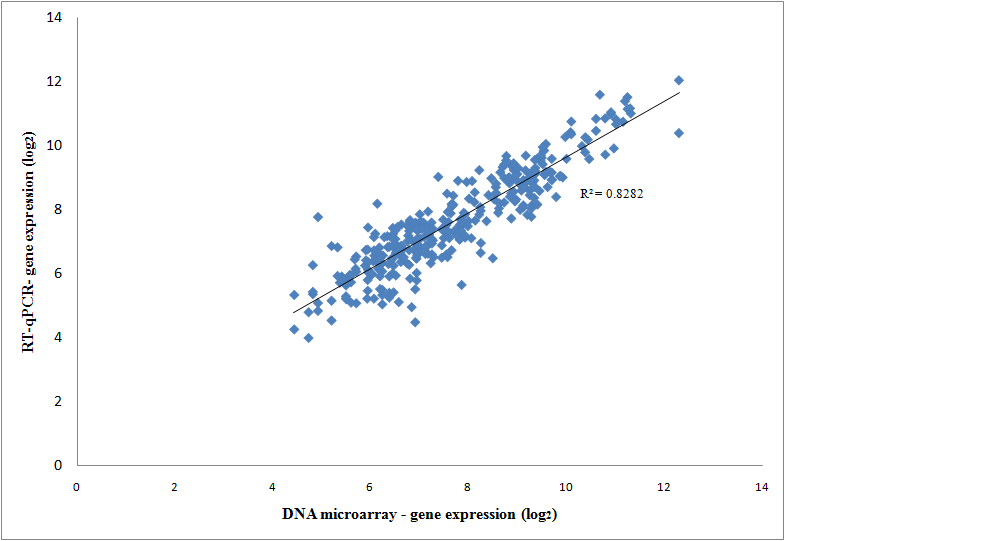

Supplement: Additional file 2: — Correlation between gene expression for the DNA microarray and the RT-qPCR. Correlation for the expression of the 14 potential marker genes for stability between the DNA microarray and the RT-qPCR. (TIFF 41 kb) [file 12896_2015_218_MOESM2_ESM.tiff]
